# The influence of hypoglycemia and hyperglycemia on the adverse outcome of COVID-19 combined with diabetes mellitus: A protocol for systematic review and meta-analysis

**DOI:** 10.1097/MD.0000000000022587

**Published:** 2020-10-30

**Authors:** Yan Yang, Xiumei Fan Dongqiong Chen, Yalin Chen, Hongyan Xie, Chunguang Xie, Lipin Ying

**Affiliations:** Chengdu University of Traditional Chinese Medicine, Affiliated Hospital of Chengdu University of Traditional Chinese Medicine, Sichuan, China.

**Keywords:** COVID-19, diabetes mellitus, hyperglycemia, hypoglycemia, protocol, systematic review and meta-analysis

## Abstract

**Background:**

COVID-19 has become a global epidemic, causing huge loss of life and property. Diabetes will affect the prognosis of COVID-19 patients in many ways. Both hyperglycemia and hypoglycemia can affect oxidative stress and lead to the release of inflammatory mediators, leading to multiple organ damage and chronic inflammation. Here, we want to know whether hyperglycemia or hypoglycemia will adversely affect patients with diabetes and COVID-19 comorbidities. This has very important practical significance for the control of blood glucose in the treatment of diabetes combined with SARS-COV-2 infection.

**Methods:**

We will search electronic databases including PubMed, EMBASE, the Cochrane Central Register of Controlled Trials (CENTRAL), Chinese Biomedical Literature Database (CBM), China National Knowledge Infrastructure (CNKI), Chinese Science and Technology Periodical Database (VIP), and Wanfang database using keywords related to COVID-19, diabetes mellitus, hyperglycemia and hypoglycemia. We will manually search gray literature, such as conference proceedings and academic degree dissertations, and trial registries. Two independent reviewers will screen studies, extract data, and evaluate risk of bias. Data analysis will be conducted using the Review Manager software version 5.3.5 and STATA4.0 software for Mac. The main outcome was the mortality of COVID-19 which was included in meta-analysis and subgroup analysis. The bias of the study was evaluated independently by NOS scale, and published by funnel chart. The sensitivity was analyzed row by row.

**Results:**

This study will provide a high-quality synthesis of hyperglycemia and hypoglycemia in patients with COVID-19 combined with diabetes mellitus. To provide evidence for clinical treatment of diabetes mellitus combined with COVID-19. And the results will be published at a peer-reviewed journal.

INPLASY registration number INPLASY 202080096.

## 1 Introduction

In December 2019, the coronavirus disease of 2019 (COVID-19) caused by SARS-CoV-2was first discovered in Wuhan, China. SARS-CoV-2 belongs to the B lineage of the beta-coronaviruses and is closely related to the SARS- CoV virus (80% homology).^[1–3]^ March 11, 2020 by the World Health Organization (who) announced as a global pandemic.^[4]^ Many early studies have found that COVID-19 patients with chronic disease such as diabetes, hypertension, cardiovascular disease are more severe and have worse prognosis.^[5–9]^ According to the preliminary data from the American Centers for Disease Control and Prevention on March 28, 2020, diabetes is the most common basic health condition among the people infected with SARS-CoV-2, about 10.9%. Furthermore, it is estimated that 32% of the patients who need to be admitted to ICU.^[10]^ Diabetes are also closely related to the high morbidity of obesity, hypertension, and cardiovascular diseases. Diabetic cardiovascular complications and diabetic renal complications are important risk factors for severe complications in patients with COVID-19. Blood glucose and diabetes are independent risk factors for mortality and morbidity in SARS patients.^[11]^ Chronic hyperglycemia leads to cell mitochondrial dysfunction and oxidative stress through a variety of ways, casing dysfunction of cell, tissue, and organs. The use of insulin or insulin secretagogues to control hyperglycemia usually leads to hypoglycemia.^[12]^ Data from 27,585 insulin-treated patients measured by a hypoglycemia assessment tool (HAT) showed that 83% of T1 diabetes patients had hypoglycemia, 14% had severe hypoglycemia, and 47% of type 2 diabetes patients had hypoglycemia, 9% Of T2D patients have severe hypoglycemia.^[13]^ A study of healthy controls and type 2 diabetes mellitus (T2DM) patients found that T2DM patients have comprehensive, systemic, and chronic blood hypercoagulability and this may due to the increased inflammation biomarkers and fibrinogen amyloid changes caused by hyperglycemia.^[14]^ More and more studies proves that the important feature of COVID-19 is coagulopathy, characterized by high D-dimer and fibrinogen concentrations with minor changes in prothrombin time and platelet count.^[15,16]^ This shows that both hyperglycemia and hypoglycemia will have adverse consequences for diabetic patients. Therefore, diabetes mellitus plays a central role in the influence of underlying diseases, metabolic disorders, immune, abnormalities, and inflammatory status on COVID-19.

At present, there is no meta-analysis on the influence of the frequency of hypoglycemia and hyperglycemia on the adverse outcome of COVID-19 combined with diabetes mellitus. The main purpose of our research is to emphasize the significance of blood glucose management in diabetes and SARS-COV-2 infections.

## 2 Methods and analysis

### 2.1 Study registration

The report of this system review plan is in accordance with the preferred report item of the system review and meta-analysis plan (PRISMA-P) guidelines.^[17,18]^ This study will be conducted in accordance with the PRISMA extension statement of NMA.^[19]^

### 2.2 Inclusion and exclusion criteria

#### 2.2.1 Population

Diabetic patients infected with SARS-CoV-2 will be included in our study. There are no restrictions on the region, gender, and age of patients.

#### 2.2.2 Intervention

This study will investigate the relationship between the frequency of hyperglycemia and hypoglycemia and adverse outcomes in patients with diabetes and SARS-COV-2 infections. Divide them into hyperglycemia group and hypoglycemia group.

#### 2.2.3 Study design

All studies of diabetes combined with COVID-19 will be included.

### 2.3 Outcomes

The number of Patients entering the ICU (Intensive Care Unit)

- Dead patients
- Course of disease
- Coagulation status

### 2.4 Study search

We will search electronic databases including PubMed, EMBASE the Cochrane Central Register of Controlled Trials (CENTRAL), Chinese Biomedical Literature Database (CBM), China National Knowledge Infrastructure (CNKI), Chinese Science and Technology Periodical Database (VIP), and Wanfang database using keywords related to COVID-19, diabetes mellitus, hyperglycemia, hypoglycemia. We will manually search gray literature, such as conference proceedings and academic degree dissertations, and trial registries. Two independent reviewers will screen studies, extract data, and evaluate risk of bias. Data analysis will be conducted using the Review Manager software version 5.3.5 and STATA14.0 software for Mac. The main outcome was the mortality of COVID-19 which was included in meta-analysis and subgroup analysis. The bias of the study was evaluated independently by NOS scale, and published by funnel chart. The sensitivity was analyzed row by row. An example of search process is presented in Table 1.

**Table 1:**
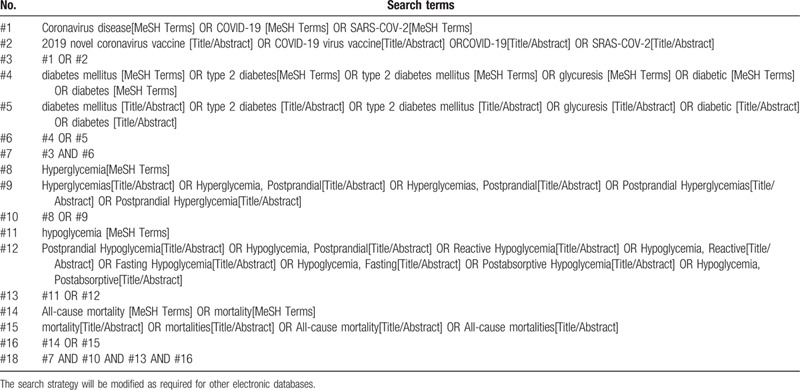
Search strategy for PubMed.

### 2.5 Study selection

**Figure 1:**
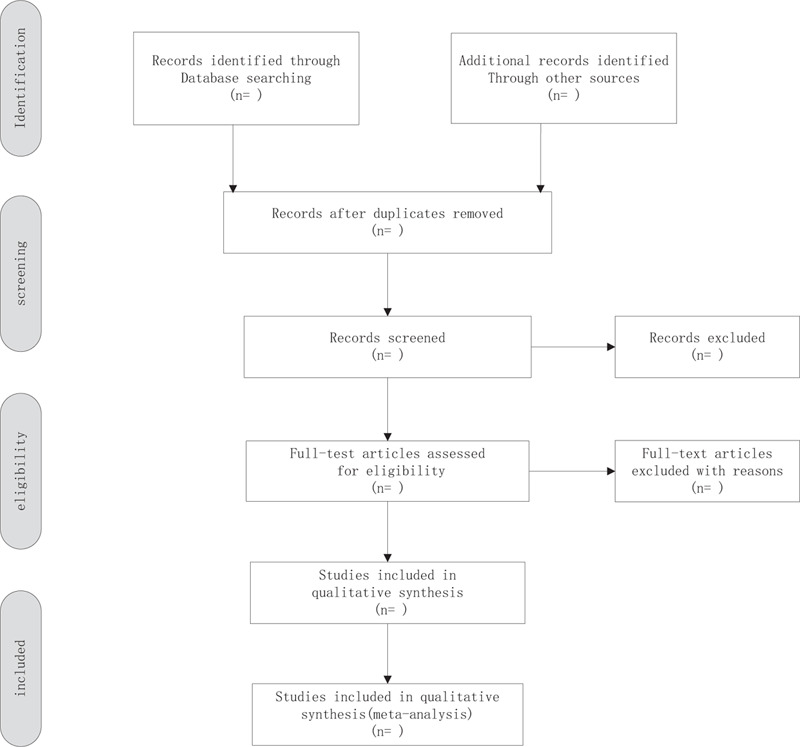
Flow chart of the study selection.

Based on the pre-determined inclusion criteria, 2 independent reviewers will evaluate all titles and abstracts to exclude papers that are not considered relevant. The remaining provisions will be included in a further assessment. Reviewers will carefully examine the full text of each potentially relevant article. The process of study identification and exclusion will be described by PRISMA flow chart. Differences in research options will be resolved through consultation. And record in Excel file (Fig. 1).

### 2.6 Data extraction and exclusion

Two researchers independently screened literature, extracted data and cross checked them. In case of any difference, it shall be settled through discussion or consultation with a third party. In the process of literature selection, the first step is to read the title. After excluding the obviously irrelevant literature, the second step is to read the abstract and the full text to determine whether to include it. If necessary, contact the author of the original study by email or phone to obtain the uncertain but very important information for this study. The content of data extraction includes:

1. Basic information: the first author, publication time, research location, sample size, sex ratio, age, research type;
2. Outcome indicators of concern;
3. Relevant elements of bias risk assessment.

### 2.7 Exclusion criteria

- Non-Chinese and English literature;
- Literature with repeated reports;
- Literature without relevant outcome indicators;
- Literature with incomplete or missing analysis data and unable to contact the original author.

### 2.8 Risk of bias assessment

Bias risk assessment included in the study: 2 researchers independently assessed the bias risk of the included study with NOS scale, and cross checked the results.

### 2.9 Data analysis

Data analysis will be conducted in Review Manager Version 5.3 and SATAT14.0 software for Mac. The risk ratio (RR) was used as the analysis statistic and 95% CI was provided. The heterogeneity of the results was analyzed by χ^2^ test (the test level was α = 0.1), and the degree of heterogeneity was determined by *I*^2^. If there is no statistical heterogeneity between the results of each study, the fixed effect model is used for meta-analysis; if there is statistical heterogeneity between the results of each study, the source of heterogeneity is further analyzed. After excluding the influence of obvious clinical heterogeneity, the random effect model is used for meta-analysis. The level of meta-analysis is set as α = 0.05. Significant clinical heterogeneity was treated by subgroup analysis or sensitivity analysis, or only descriptive analysis.

### 2.10 Investigation of heterogeneity

If there is substantial heterogeneity between studies, then we will conduct subgroup analysis to explore the heterogeneity. To avoid post hoc analysis, the subgroup analysis will be conducted according to diabetes complications. To further improve the reliability of subgroup analysis, we will evaluate the credibility of our subgroup analysis according to the guidance for credible subgroup analysis. If there are enough studies included, then meta-regression will be conducted to further explore the heterogeneity. Those subgroup effects that occur simultaneously in subgroup analysis and regression analysis will be considered credible.

### 2.11 Sensitivity analysis

Draw funnel chart for Patients entering the ICU indicators of diabetes and non-diabetes COVID-19 patients. To ensure the stability of the results, we will conduct sensitivity analysis of the results by excluding each of the studies included in the analysis one by one, then re-analyzing the results, and comparing the differences between the re-obtained results and the original results. In this way, we will be able to assess the impact of individual studies on overall outcomes and their robustness.

### 2.12 Reporting bias assessment

The integrity of the studies is an important factor affecting the accuracy of the results and conclusions of meta-analysis. The integrity of the included studies is mainly measured by reporting bias, of which publication bias is the most common. Therefore, this study will identify report bias by publication bias assessment. A funnel plot will be drawn to investigate the publication bias. Funnel plot will be asymmetric when publication bias exists, such as when research with small sample and no statistically significant results are not published. The more obvious the asymmetry of funnel plot is, the more likely there is publication bias.^[20]^ And then Egger test will be conducted for statistical assessment the publication bias. The publication bias is considered to exist if *P* <  .05.^[21]^

### 2.13 Patient and public involvement

No patients or public will participate in the study.

### 2.14 Ethics and dissemination

Since confidential patient data will not be involved in this study, formal ethics approval is not required. The frame- work of the PRISMA statements for NMA will be applied to guide review authors to perform this study. The results will be disseminated by a peer-reviewed publication.

## 3 Discussion

Like the evolution of other species, viruses are constantly changing and developing in nature. The outbreak and spread of SARS-CoV, SARS-CoV-2 or MERS-COV are great survival challenge for us humans. The outbreak and spread of SARS-CoV, SARS-CoV-2 or MERS-COV is a huge survival challenge for us humans. For diabetes, blood glucose control is always fluctuating due to various reasons. The occurrence of hyperglycemia and hypoglycemia has a huge impact on the patient's organs. Especially when combined with SARS-C0V-2 infection may cause greater damage. In this case, our goal is to provide reliable evidence for blood glucose control in diabetic patients with COVID-19 and provide guidance for clinical treatment.

## Author contributions

If any modification is required, we will update our protocol to include any changes in the entire research process.

**Conceptualization:** Yan Yang, Xiumei Fan.

**Data curation:** Yan Yang, Xiumei Fan, Dongqiong Chen.

**Formal analysis:** Yan Yang, Dongqiong Chen.

**Funding acquisition:** Yalin Chen.

**Methodology:** Yan Yang, Dongqiong Chen.

**Resources:** Hongyan Xie, Lipin Ying.

**Software:** Xiumei Fan, Dongqiong Chen.

**Supervision:** Lipin Ying.

**Writing – original draft:** Yan Yang.

**Writing – review & editing:** Yan Yang, Chunguang Xie.
